# The efficacy and safety of subcutaneous continuous local infiltration analgesia with ropivacaine in patients undergoing total knee arthroplasty: a comparative study

**DOI:** 10.1186/s12891-023-06263-7

**Published:** 2023-03-22

**Authors:** Maad F. Al-Saati, Sadiq I. Alaqaili, Farah A. Alshammari, Mohamed A. N. ElRaei, Alia A. Albaiz, Daniel Tushinski, Omar A. Al-Mohrej

**Affiliations:** 1 Section of Orthopedic Surgery, Department of Surgery, King Abdullah Bin Abdulaziz University Hospital, Princess Nourah Bint Abdul Rahman University, Riyadh, Saudi Arabia; 2 Department of Orthopedic Surgery, Dammam Medical Complex, Dammam, Saudi Arabia; 3 College of Medicine, Princess Nourah Bint Andulrahman University, Riyadh, Saudi Arabia; 4 Department of Epidemiology and Biostatistics, Health Science Research Center, Princess Nourah Bint Abdulrahman University, Riyadh, Saudi Arabia; 5 Division of Orthopaedics, Department of Surgery, McMaster University, Hamilton, ON Canada

**Keywords:** Pain control, Total knee arthroplasty, Opioid reduction, Continuous local infiltration analgesia

## Abstract

**Background:**

Continuous local infiltration analgesia (CLIA) can be administered via intraarticular or periarticular techniques in patients undergoing total knee arthroplasty (TKA). The purpose of this investigation was to retrospectively report a single-center experience of epidural analgesia with subcutaneous CLIA versus epidural analgesia without CLIA among patients undergoing TKA.

**Methods:**

This single-center retrospective study was conducted in Saudi Arabia. From January 01, 2014, to December 30, 2020, medical records of all patients who underwent TKA were reviewed. Patients who received subcutaneous CLIA with epidural analgesia were assigned to the intervention group, whereas those who received epidural analgesia without subcutaneous CLIA were assigned to the control group. The efficacy endpoints included: (i) postoperative pain scores at 24 h, 48 h, 72 h, and 3 months; (ii) postoperative opioid consumption at 24 h, 48 h, 72 h, and 24–72 h (cumulative); (iii) length of hospital stay; and (iv) postoperative functional recovery of the knee 3 months post-operation, according to the Knee Injury and Osteoarthritis Outcome Score.

**Results:**

At rest and during mobilization, the CLIA group (*n* = 28) achieved significantly lower postoperative pain scores 24 h, 48 h, 72 h, and 3 months post-operation than the non-CLIA group (*n* = 35). Subgroup analysis revealed that the CLIA group achieved significantly less opioid consumption 24 h and 48 h post-operation than the non-CLIA group. There was no difference between the groups regarding the length of hospital stay or functional scores 3 months post-operation. There was no significant difference between the groups regarding the rate of wound infection, other infections, and readmission within 30 days.

**Conclusion:**

Subcutaneous CLIA is a technically feasible and safe procedure without major adverse events but with reduced postoperative pain scores (at rest and during mobilization) and opioid consumption. Additional larger studies are warranted to confirm our results. Moreover, a head-to-head comparison between subcutaneous CLIA and periarticular or intraarticular CLIA is an interesting prospective investigation.

**Supplementary Information:**

The online version contains supplementary material available at 10.1186/s12891-023-06263-7.

## Introduction

Total knee arthroplasty (TKA) is one of the most commonly performed orthopedic procedures. TKA enhances the quality of life of patients to achieve a high degree of functionality [1]. The optimal management of postoperative pain among patients who undergo TKA remains a topic of contention as 30% to 60% of patients undergoing TKA experience moderate and severe postoperative pain, with pain at its highest severity 3–6 h after surgery and persisting for up to 3 days post-operation [2, 3].

Numerous pain relief modalities exist to control postoperative pain following TKA but no superior treatment modalities have been identified [3]. Such modalities generally comprise epidural anesthesia, peripheral nerve block, local infiltration analgesia (LIA), opioids, patient-controlled analgesia, and multimodal analgesia [3–5]. Zhang et al. showed that LIA offers good pain control and promotes early mobilization following TKA. Nonetheless, the pain-relieving effects of LIA disappear within the first 24 h. Consequently, efforts have been made to extend the duration of pain relief via pump-based continuous local infiltration analgesia (CLIA) [3].

LIA and CLIA can be administered via intraarticular or periarticular techniques in patients undergoing TKA [6]. To the best of our knowledge, the analgesic utility and safety of subcutaneous CLIA among patients undergoing TKA have been examined in several studies [7, 8]. However, none was done in Saudi Arabia, or middle east region. Thus, the purpose of this investigation was to retrospectively report our single-center experience of epidural analgesia with subcutaneous CLIA versus epidural analgesia with no subcutaneous CLIA among patients undergoing TKA.

## Materials and methods

This retrospective study was conducted at King Abdullah bin Abdulaziz University Hospital, Riyadh, Saudi Arabia. The study protocol was performed in accordance with the 1964 Declaration of Helsinki. The Institutional Review Board (IRB) of Princess Nourah bin Abdulrahman University, Riyadh, Saudi Arabia has determined that the project poses no more than minimal risk to the participants. Therefore, the proposal has been waived.

From January 01, 2014, to December 30, 2020, the medical records of all patients who underwent TKA were reviewed. The inclusion criteria were as follows: (i) patients who underwent unilateral TKA; (ii) patients who received subcutaneous CLIA with epidural analgesia or standard of care analgesia (i.e., epidural analgesia alone); (iii) age less than 80 years; (iv) American Society of Anesthesiologists (ASA) physical status I–III; (v) body mass index < 40 kg/m^2^; (vi) no known allergies to ropivacaine; and (vii) the drug used for subcutaneous CLIA was ropivacaine. The major exclusion criteria were as follows: (i) patients who underwent bilateral TKA with/without additional procedures; (ii) patients who received periarticular or intraarticular CLIA; (iii) age > 80 years; (iv) ASA physical status IV–V; (v) known allergy to ropivacaine; and (vi) the drug used for subcutaneous CLIA was something other than ropivacaine. The sample size included all patients who met the eligibility criteria during the study period. Therefore, the sample size was not calculated a priori.

Patients were divided into two groups: the intervention/CLIA group received subcutaneous CLIA plus epidural analgesia, whereas the control/non-CLIA group received epidural analgesia without subcutaneous CLIA. All TKA procedures were performed by a single surgeon (MFA). TKA was performed using the medial parapatellar approach with a 250-mmHg tourniquet under epidural anesthesia. The solution used for CLIA was 200 mg of ropivacaine (2 mg/ml). A drainage tube was placed laterally on the prosthesis in each knee. The CLIA was administered subcutaneously. No patient received any regional nerve block or epidural block during the perioperative period. Subcutaneous CLIA delivers medication at constant flow rate of 5 ml/hr for 48 h. After surgery, participants routinely received 40 mg of parecoxib every 12 h and 650 mg of acetaminophen every 8 h. The rescue analgesia treatment included morphine and tramadol. The control group post operative pain protocol was as following: Tramadol 50 mg IV every 6 h over 20 min for pain score 6–10, Paracetamol 1 gm IV every 6 h’ pain score 3 for 2 days, Paracetamol 1 gm p.o every 6-h PRN for pain score 3.

Preoperative data, including age, sex, and coexisting morbidities, were collected. The study outcomes included efficacy and safety endpoints. The efficacy endpoints included: (i) postoperative pain scores at 24 h, 48 h, 72 h, and 3 months; (ii) postoperative opioid consumption at 24 h, 48 h, 72 h, and 24–72 h (cumulative); (iii) length of hospital stay; and (iv) postoperative functional recovery of the knee 3 months post-operation. Postoperative pain scores were evaluated according to the 10-point visual analog scale (VAS), where “0” represents no pain at all and “10” represents the maximal pain possible. Opioid consumption was evaluated according to the quantitative milligram administered and converted to milligram morphine equivalent (MME). The length of hospital stay was counted from postoperative day 0 until the day of discharge. Postoperative functional recovery of the knee was evaluated according to the Knee Injury and Osteoarthritis Outcome Score (KOOS) [9]. The complication endpoints included the rates of wound infection, other infections, deep vein thrombosis, pulmonary embolism, cardiac arrhythmia, and readmission within 30 days.

Data were analyzed using Statistical Package for Social Sciences (SPSS) version 24.0. Categorical data were presented as numbers and percentages. Numerical data were presented as means ± standard deviations, as well as mean difference (MD) and 95% confidence interval (CI). The chi-square test of independence was used for the univariate analysis of categorical data. Student’s t-test was used for the univariate analysis of numerical data. All statistical analyses were two-tailed. For all purposes, a *p*-value < 0.05 was considered statistically significant.

## Results

Sixty-three patients (*n* = 63) were included in the study. There were 28 and 35 patients allocated to the CLIA and non-CLIA groups, respectively. The mean age in CLIA and non-CLIA groups was 63.93 ± 6.51 and 61.97 ± 9.8 years (*p* = 0.368), respectively, and the body mass index was 35.74 ± 5.89 and 36.5 ± 5.05 kg/m^2^ (*p* = 0.583), respectively. There were no significant differences in preoperative characteristics between the groups (Table 1).

**Table 1:** Baseline (preoperative) characteristics of the patients

Characteristic	Pump (*n* = 28)		No pump (*n* = 35)		*p*-value
	n	%	n	%	
Female gender	24	85.7	33	94.3	0.249
Congestive heart failure	0	0	1	2.9	0.367
Ischemic heart disease	2	7.1	6	17.1	0.367
Hypertension	23	82.1	24	68.6	0.219
Asthma	6	21.9	2	5.7	0.063
Chronic renal failure	1	3.6	1	2.9	0.872
Dyslipidemia	4	14.3	5	14.3	1
Hypothyroidism	4	14.3	2	5.7	0.249

At rest, the CLIA group achieved significantly lower postoperative pain scores 24 h (MD =  − 2.88, 95% CI [–4.4, –1.36], *p* < 0.001), 48 h (MD = –2.5, 95% CI [–3.86, –1.14], *p* = 0.001), 72 h (MD = –1.98, 95% CI [–3.38, –0.58], *p* = 0.006), and 3 months (MD = –0.59, 95% CI [–0.96, –0.21], *p* = 0.003) post-operation than the non-CLIA group. During mobilization, the CLIA group achieved significantly lower postoperative pain scores at 24 h (MD =  − 2.62, 95% CI [–4.02, –1.22], *p* < 0.001), 48 h (MD = –3.17, 95% CI [–4.15, –1.83], *p* < 0.001), 72 h (MD = –2.78, 95% CI [–4.15, –1.37], *p* < 0.001), and 3 months (MD = –0.514, 95% CI [–0.92, –0.11], *p* = 0.014) than the non-CLIA group (Table 2).

**Table 2:** Visual analogue scale (VAS) postoperative pain scores

Time point	Pump (*n* = 28)		No pump (*n* = 35)		MD	95% CI		*p*-value
	Mean	SD	Mean	SD		Lower limit	Higher limit	
At rest, 12 h	4.68	2.64	5.89	4.02	-1.207	-2.970	0.556	0.176
At rest, 24 h	3.75	2.62	6.63	3.26	-2.879	-4.397	-1.360	< 0.001
At rest, 48 h	3.36	2.38	5.86	2.90	-2.500	-3.860	-1.140	0.001
At rest, 72 h	2.96	2.17	4.94	3.14	-1.979	-3.375	-0.582	0.006
At rest, 3 months	1.07	0.38	1.66	0.94	-0.586	-0.963	-0.209	0.003
On mobilization, 12 h	5.61	2.74	6.23	4.17	-0.621	-2.452	1.209	0.500
On mobilization, 24 h	4.75	2.50	7.37	2.94	-2.621	-4.019	-1.224	< 0.001
On mobilization, 48 h	3.86	2.65	7.03	2.64	-3.171	-4.512	-1.831	< 0.001
On mobilization, 72 h	3.36	2.41	6.11	2.99	-2.757	-4.149	-1.365	< 0.001
On mobilization, 3 months	1.14	0.59	1.66	0.94	-0.514	-0.921	-0.107	0.014

*CI* Confidence interval, *MD* Mean difference, *SD* Standard deviation

The CLIA group achieved significantly less total opioid consumption during hospital stay than the non-CLIA group (MD = –6.34 MME, 95% CI [–11.24, –1.62], *p* = 0.01). Subgroup analysis revealed that the CLIA group achieved significantly less opioid consumption 24 h (MD = –3.25 MME, 95% CI [–5.52, –0.98], *p* = 0.006) and 48 h (MD = –1.93 MME, 95% CI [–3.62, –0.24], *p* = 0.026) post-operation than the non-CLIA group (Table 3). There was no difference between the groups regarding the length of hospital stay (MD = 0.08 days, 95% CI [–0.75, 0.91], *p* = 0.85).

**Table 3:** Postoperative opioid consumption according to morphine milligram equivalent (MME)

Postoperative time point	Pump (*n* = 28)		No pump (*n* = 35)		MD	95% CI		*p*-value
	Mean	SD	Mean	SD		Lower limit	Higher limit	
24 h	1.61	2.740	4.86	5.489	-3.250	1.137	-0.976	0.006
48 h	1.07	2.493	3.00	3.873	-1.929	0.845	-0.238	0.026
72 h	0.89	2.378	2.14	3.695	-1.250	0.806	0.362	0.126
24–72 h	3.57	7.052	10.00	11.048	-6.429	2.406	-1.618	0.010

*CI* Confidence interval, *MD* Mean difference, *SD* Standard deviation

There were no significant differences between the groups for total and domain-specific items at both the preoperative and postoperative time points (Table 4). None of the patients in either group experienced intraoperative complications (Table 5). Moreover, no patients in either group experienced deep vein thrombosis or pulmonary embolism. There was no significant difference between the groups regarding the rate of wound infection (0% vs. 2.9%, *p* = 0.37), other infections (3.6% vs. 5.7%, *p* = 0.37), and readmission within 30 days (0% vs. 11.4%, *p* = 0.07). Of note, no complications were seen due to ropivacaine (continuous infiltration/overdosage of ropivacaine) in CLIA group.

**Table 4:** Knee Injury and Osteoarthritis Outcome Score (KOOS) before and after total knee arthroplasty

Time point/item	Pump (*n* = 28)		No pump (*n* = 35)		MD	95% CI		*p*-value
	Mean	SD	Mean	SD		Lower limit	Higher limit	
Preoperative/symptoms	42.93	24.50	42.71	24.71	0.214	-12.266	12.695	0.973
Preoperative/pain	29.11	20.52	26.74	19.94	2.364	-7.876	12.605	0.646
Preoperative/function, daily living	38.39	21.32	44.94	19.67	-6.550	-16.900	3.800	0.211
Preoperative/function, sports, and recreational activities	51.79	39.87	64.77	41.12	-12.986	-33.554	7.582	0.212
Preoperative/quality of life	26.29	15.75	27.46	17.21	-1.171	-9.577	7.235	0.781
Postoperative/symptoms	87.54	11.38	85.91	12.18	1.621	-4.377	7.620	0.591
Postoperative/pain	87.18	13.42	91.09	10.86	-3.907	-10.020	2.206	0.206
Postoperative/function, daily living	81.86	17.76	88.11	12.27	-6.257	-13.837	1.322	0.104
Postoperative/function, sports, and recreational activities	67.29	37.59	73.74	35.30	-6.457	-24.876	11.962	0.486
Postoperative/quality of life	83.86	14.52	78.63	17.89	5.229	-3.127	13.584	0.216

*CI *Confidence interval, *MD* Mean difference, *SD* Standard deviation

**Table 5:** Postoperative adverse events

Postoperative time point	Pump (*n* = 28)		No pump (*n* = 35)		*p*-value
	n	%	n	%	
Wound infection	0	0	1	2.9	0.367
Other infection	1	3.6	2	5.7	0.691
Pneumonia	2	7.1	0	0	0.108
Delirium	1	3.6	0	0	0.260
Anemia requiring transfusion	2	7.1	0	0	0.108
Readmission within 30 days	0	0	4	114	0.065
Cardiac dysfunction	1	3.6	2	5.7	0.691

## Discussion

This retrospective study compared the analgesic efficacy and safety of subcutaneous CLIA versus epidural analgesia among 63 patients who underwent TKA. Our results showed that subcutaneous CLIA is technically feasible and safe, without major adverse events. Moreover, we revealed that subcutaneous CLIA was correlated with reduced postoperative pain scores (at rest and during mobilization) and opioid consumption. However, there was no difference between the groups regarding the mean length of hospital stay or functional recovery according to the KOOS questionnaire, 3 months post-operation.

Nearly 55% and 70% of postoperative pain following TKA occurs at rest and during mobilization, respectively [2]. Our investigation demonstrated that postoperative pain scores were lower in the subcutaneous CLIA group than in the epidural analgesia group. This observation was true for both at rest and during mobilization. Our results of reduced postoperative pain scores were in line with earlier studies that demonstrated the superior efficacy of ropivacaine to saline for subcutaneous CILA [7, 8]. Moreover, in our study, these reductions in postoperative pain scores were not only statistically significant but also clinically meaningful. The minimal clinically important difference for improved postoperative analgesia was defined by Myles et al. as a reduction of 1 point out of the 10-point (or 10 points out of 100-mm) pain VAS [10]. The substantial improvement in postoperative pain scores was corroborated by the reduced postoperative consumption of opioids during the first 48 h post-operation. Postoperative opioid prescription is a major concern among patients with TKA and a high-risk factor for chronic opioid addiction [11, 12]. Thus, analgesic interventions at any point in the perioperative period that reduce opioid consumption are highly warranted. Based on our results, subcutaneous CLIA appears to achieve this goal.

Postoperative pain is a major reason for the increased length of hospitalization after TKA [4, 13]. Our results showed that there was no difference in hospital stay between the groups, even though postoperative pain scores and opioid consumption were significantly reduced in the subcutaneous CLIA group than in the control group. One potential reason for the lack of statistically significant differences in hospital stay can be ascribed to the hospital protocol of discharging patients on postoperative day 5.

Functional recovery improved in both groups after TKA (within-group changes comparing postoperative and preoperative values). However, there was no significant difference between the groups in KOOS scores 3 months post-operation (long-term) in all domains. Eckhard et al. [14] reported that the minimal clinically important difference values for pain, function-daily living, and quality of life are 12.5, 15.2, and 8, respectively, in the KOOS-12 questionnaire.

LIA and CLIA can be administered via intraarticular or periarticular techniques in patients undergoing TKA [6]. The optimal method of administration is yet to be determined [6], although periarticular administration appears to be more beneficial than the intraarticular method [6, 15]. Knee infection and drainage are key adverse events of CLIA [16]. Herein, we elected to use an alternative method of CLIA (subcutaneous administration) to maximize analgesic benefits and minimize the hazards of knee injection and drainage-related aftermath. Our results showed that subcutaneous CLIA is feasible and technically safe without major side effects.

Future research should include conducting a large multicenter prospective study to increase the power of the results on the analgesic utility of subcutaneous CLIA among patients undergoing TKA. Additional research may include direct head-to-head comparisons between subcutaneous CLIA and intraarticular or periarticular CLIA among patients undergoing TKA and identifying the subgroup of patients with TKA who are likely to benefit the most from the administration of subcutaneous CLIA.

Our study had several strengths. To the best of our knowledge, this is the first study to explore the feasibility, safety, and analgesic efficacy of subcutaneous CLIA in patients undergoing TKA. Additional strengths included that all operations were performed by a single surgeon to minimize procedure-related bias and the measurement of efficacy outcomes at different short- and long-term time points. Nonetheless, our investigation was not without limitations. Key limitations included the small sample size, retrospective study design, single-center experience, and recall bias associated with the measurement of efficacy endpoints 3 months post-operation. Although CLIA was given to all patients fitted the inclusion and exclusion criteria during the study period. Yet, selection bias cannot be fully eliminated.

## Conclusion

Among patients undergoing TKA, subcutaneous CLIA is technically feasible and safe, without major adverse events. Subcutaneous CLIA was correlated with lower postoperative pain scores (at rest and during mobilization) and less opioid consumption than epidural analgesia. There was no difference between the groups in terms of hospital stay or functional recovery 3 months post-operation. Additional larger studies are warranted to confirm our results. Moreover, head-to-head comparison between subcutaneous CLIA and periarticular or intraarticular CLIA is an interesting prospective investigation.

## Supplementary Information

**Additional file 1.**

## Data Availability

The datasets used and/or analyzed during the current study are available from the corresponding author on reasonable request.

## Abbreviations

TKA: Total knee arthroplasty
LIA: Local infiltration analgesia
CLIA: Continuous local infiltration analgesia
KOOS: Knee Injury and Osteoarthritis Outcome Score
