# Supplementary material for: The efficacy and safety of subcutaneous continuous local infiltration analgesia with ropivacaine in patients undergoing total knee arthroplasty: a comparative study
Source: BMC Musculoskelet Disord. 2023 Mar 22;24:214. doi: 10.1186/s12891-023-06263-7 (PMC10032015; doi:10.1186/s12891-023-06263-7)
Supplement: Supplementary file 1 — Additional file 1. [file 12891_2023_6263_MOESM1_ESM.docx]

Self-contained, portable, ready-to-use infusion system, pre-filled with 250 ml 0.2% Ropivacaine for postoperative pain. Ropivacaine Readyfusor delivers medication at constant flow rate of 5ml/hr providing 48 hours of pain treatment.

Technique:

1 Catheter Placement

• Remove the protective cover of the introducer needle.

• Insert the introducer needle from inside the surgical site from the suprapatellar pouch to laterally between 5 cm and 7 cm from the edge of the incision.

The luer cap should be fixed to the catheter prior to entry.

• Remove the introducer needle from the sheath.

• Insert the catheter as indicated.

Post-operative pain management using local anaesthetics after TKA when the catheter was placed intraarticular has been associated with pain opioid use reduction.

Following expert opinion and clinical literature ideal catheter placement is recommended as follows: the fenestrated catheter (bleu line on figure 1) is inserted laterally through the capsule into the joint. The proximal part of the fenestrated area is placed into the medial gutter and passes the medial side of the femoral component of the prosthesis. When reaching the plastic component of the prosthesis, the catheter is guided over the tibial component of the prosthesis from the medial to the lateral side.

If a drain would be applied as well, it is advised not to place the fenestrated part of the catheter and drain immediately adjacent to one another in order to avoid nullifying the action of ropivacaine in the surgical site.

• Remove the sheath while holding onto the catheter. Split the sheath when it is completely withdrawn. 2 Securing Catheter & Wound Closure

• Secure the catheter at entry, loop excessive catheter length twice and secure it with steri-strips 3-4 cm distant from the entry point of the catheter . The entry point of the catheter should not be sutured.

Properly securing the catheter is of high importance to avoid catheter migration. In case the patient will be ambulant at an early stage after surgery, please consider additional securing techniques to fix the catheter (e.g. using tissue glue at the tunnel entrance of the catheter).

• Activate the Ropivacaine Readyfusor at this time to ensure no delay when connecting later (see below).

It may take 3-5 minutes for the fluid to move fully through the end of the line.

• Close the surgical site as per standard of care.

• After cleaning the wound area, apply a transparent film dressing patch over the secured looped catheter, separately from wound sutures and dressings.

3 Immediate Onset of Action and Ropivacaine Readyfusor connection

• Remove the luer cap from the catheter.

• Administer a loading dose of 10 ml of a local anaesthetic (10ml 1% recommended of e.g. ropivacaine, bupivacaine or levobupivacaine) as a bolus through the catheter.

Pre-infusion wound tissue local anaesthetic infiltration is an alternative to be considered.4 When choosing this option, it is recommended to use the same volume and concentration of local anaesthetics (10ml of 1%). This type of infiltration should be performed prior to closing the wound.

• Remove the luer lock cap at the end of the administration line of the Ropivacaine Readyfusor after fluid has reached tube end.

• Connect the administration line of the Ropivacaine Readyfusor with the luer lock of the catheter.
